# Efficacy of Autologous Platelet-Rich Plasma Injections for Treating Plantar Fasciitis

**DOI:** 10.7759/cureus.72208

**Published:** 2024-10-23

**Authors:** Waseem Ahmad, Rahim Ullah, Zia Ullah, Arsalan Shah Roghani, Muhammad Maaz Raza, Rao Erbaz Hassan, Moiz I Khan, Hafiz Mursalin Khan, Mustafa Faraj, Irfan Khan

**Affiliations:** 1 Trauma and Orthopedics, Mufti Mehmod Memorial Teaching Hospital, Dera Ismail Khan, PAK; 2 Surgery, Hayatabad Medical Complex Medical Teaching Institute (MTI), Peshawar, PAK; 3 Trauma and Orthopedics, Khyber Teaching Hospital Medical Teaching Institute (MTI), Peshawar, PAK; 4 Accident and Emergency, District Headquarter Teaching Hospital, Dera Ismail Khan, PAK; 5 Plastic Surgery, Lady Reading Hospital Medical Teaching Institute (MTI), Peshawar, PAK; 6 Clinical Research, Wayne State University Detroit Medical Center, Detroit, USA

**Keywords:** autologous platelet-rich plasma, pain, plantar fasciitis, platelet-rich plasma (prp), visual analog scale (vas)

## Abstract

Introduction

Plantar fasciitis, a common cause of heel pain, is often treated conservatively. Platelet-rich plasma (PRP) injections have emerged as a promising treatment option. This study aimed to evaluate the efficacy of autologous PRP injections for plantar fasciitis. The objective of this study was to assess the effectiveness of autologous PRP injections delivered at the plantar fascia origin in reducing pain (measured by the Visual Analog Scale (VAS) pain intensity score) in patients with plantar fasciitis.

Materials and methods

A prospective cohort study was conducted at the Department of Orthopedics, Hayatabad Medical Complex, Peshawar. Patients with plantar fasciitis, a VAS pain score ≥ 7, and failure of conservative treatment were included. A single injection of autologous PRP was administered to the plantar fascia. Pain reduction was assessed using the VAS score at a 12-week follow-up.

Result

The study included 163 patients. Success was achieved in 80.3% of cases, showing that autologous PRP injection resulted in significant pain improvement in patients with plantar fasciitis (p≤0.05). An increased likelihood of successful treatment was observed in individuals with symptoms lasting less than 12 months. No other variables (gender, age group, residence, literacy) significantly impacted treatment efficacy.

Conclusion

Autologous PRP injections may be a promising treatment option for plantar fasciitis, particularly when administered early. Further research is needed to validate these findings and explore optimal patient selection criteria.

## Introduction

Plantar fasciitis is one of the leading causes of heel pain [1]. Its pathophysiology remains poorly understood and is thought to be multifactorial. Established risk factors include reduced dorsiflexion, obesity, advanced age, poor footwear, and prolonged weight-bearing [2]. While plantar fasciitis typically resolves spontaneously, rehabilitation may extend over several months. However, chronic and debilitating pain can significantly impact healthcare and quality of life [3,4]. Conservative treatment options include rest, stretching exercises, orthotics, night splints, heel cups, and nonsteroidal anti-inflammatory medication (NSAIDs). These interventions resolve approximately 80% of cases [5]. Nevertheless, cases unresponsive to conservative measures require invasive procedures. Intralesional steroid infiltration is commonly used for chronic plantar fasciitis [6]. Although effective, conservative treatment offers only short-term pain relief and may lead to complications such as application site infections, plantar fascia rupture, and fat pad atrophy [7]. Platelet-rich plasma (PRP), obtained through centrifugation of whole blood, is an emerging therapy for plantar fasciitis. PRP contains a higher concentration of platelets than whole blood. Platelets released from PRP stimulate and accelerate the natural tissue healing process by releasing various growth factors and cytokines [8]. Current evidence indicates promising results for PRP in treating plantar fasciitis [9-11]. However, the efficacy of autologous PRP injection for plantar fasciitis specifically in our local population has not been studied. Therefore, we conducted this prospective case series to evaluate the efficacy of autologous PRP injections in reducing pain in patients with plantar fasciitis. We evaluated the treatment's effectiveness by measuring pain reduction using the Visual Analog Scale (VAS) pain intensity score. We also stratified the treatment effect by demographic variables to highlight if there is any association.

## Materials and methods

This prospective cohort study was conducted at the Department of Orthopedics, Hayatabad Medical Complex, Peshawar. The sample size was calculated as 163 using the WHO calculator, with a 95% confidence interval, a 5% margin of error, and an 88% success rate for PRP injections in plantar fasciitis [12], and non-probability consecutive sampling was employed. Patients aged 18 or older with heel pain primarily localized to the plantar aspect were included if they had experienced persistent pain despite at least 12 weeks of conservative treatment (rest, nonsteroidal anti-inflammatory medication, eccentric stretching exercises, orthotics, night splints, and heel cups), had a VAS pain intensity score of seven or higher, and had a platelet count exceeding 250 per microliter (μL). Patients were excluded if they had atypical plantar fasciitis (pressure due to any lesion near or within the plantar fascia), severe diabetes mellitus (HbA1C >10%), peripheral vascular disease, a history of plantar fascia surgery, any other chronic inflammatory joint disease such as rheumatoid arthritis, a bleeding disorder, or any other invasive therapy for plantar fasciitis such as local corticosteroid injection, or if their pain was not localized to the heel. The Hayatabad Medical Complex Research Hospital and Ethical Committee reviewed the article in accordance with the Declaration of Helsinki (2013) and found it to meet the requirements and be approved (approval number: 1412). After securing ethics committee approval, researchers recruited eligible patients who provided informed consent.

A comprehensive evaluation, including detailed history, clinical examination, and baseline investigations, was then performed. All participants had prior diagnoses of plantar fasciitis confirmed by physical examination (pain upon palpation of the medial calcaneal tubercle at the plantar fascial insertion), X-rays (presence of a plantar calcaneal spur), and ultrasonography (showing fascial thickening exceeding 4.5 mm, hypoechogenicity, and edema at the insertion site, along with blurring of the fascia-soft tissue interface). Pre-intervention VAS pain intensity scores were also recorded for every patient.

All patients stopped taking NSAIDs two weeks before the injection. A single surgeon performed the PRP injections using a sterile technique. To prepare the PRP, 15-20 ml of blood was drawn from each patient into a syringe containing 2 ml of sodium citrate. The blood was then processed through a two-step centrifugation using a desktop centrifuge. The first spin, at 200 g for 10 minutes at 1,700 revolutions per minute (rpm), separated the plasma from other blood components, while the second spin, at 300 g for 10 minutes at 3,500 rpm, separated the PRP from platelet-poor plasma. The PRP was collected in a syringe, and just before injection, 1-2 ml of calcium gluconate was added to activate the platelets. Approximately 2-4 ml of the autologous PRP was injected into the center of the heel using a medial approach, without local anesthesia. The injection site was located about one finger-breath above the arch of the foot, aligned with the back edge of the medial malleolus. The complications, such as post-injection allergic reaction, persistent injection site pain lasting more than five days, or injection site infection, were recorded immediately after the injection and during follow-ups.

After the injection, patients were immediately allowed to walk but advised to avoid activities that excessively stretch the plantar fascia, such as running, jogging, or hopping, for four weeks. No physiotherapy was included in the recovery plan. Patients were followed up after one week in the outpatient department for any complications, then monthly via telephone interview for two months, and an outpatient appointment scheduled at 12 weeks for the final VAS score. Ice packs were recommended for injection site pain, and NSAIDs were prescribed as needed for up to three days. The pain was assessed at the 12-week mark using a VAS pain score. The VAS is a standard pain assessment tool widely used in clinical settings. It measures pain intensity on a 0 to 10 scale, with 0 indicating no pain and 10 representing the worst pain imaginable [13]. A successful outcome was defined as a VAS score of three or lower at the three-month follow-up. Patients who missed follow-up appointments were excluded from the study. To isolate the effects of PRP injection, no other treatments were administered during the study period.

SPSS Statistics version 24.0 (IBM Corp. Released 2016. IBM SPSS Statistics for Windows, Version 24.0. Armonk, NY: IBM Corp.) was used to analyze the data. Means and standard deviations were calculated for continuous variables like age and pain duration. Categorical variables, such as gender, age groups, residential status, literacy, disease duration, and treatment success, were presented as frequencies and percentages. A paired T-test was used for pre- and post-intervention analysis of VAS scores, and the chi-square test was used to assess the association between these variables and treatment response. A p-value of less than 0.05 was considered statistically significant at a 95% confidence interval.

## Results

The study included 168 patients with plantar fasciitis. Five were lost to follow-up, leaving 163 participants. The gender distribution was 39.3% male and 60.7% female. The mean age was 42.5 ± 6.5 years, with 63.2% aged under 50 years. The mean symptom duration was 8.4 ± 4.6 months. Illiteracy was observed in 28.8%, and 52.1% resided in urban areas. About 34.4% of patients had symptoms for over 12 months. Success in treating plantar fasciitis was achieved in 80.3% of cases (Table 1). Patients who didn't improve after 12 weeks were offered alternative treatments such as laser therapy and fasciotomy and were not further followed.

**Table 1:** Demographic characteristics of study participants SD: standard deviation

Characteristics		Values
Number of patients		163 (100%)
Gender	Males	64 (39.3%)
	Females	99 (60.7%)
Age, years, mean ± SD		42.5 ± 6.5 (range: 27-69 years)
Age range	>50 years	60 (36.8%)
	<50 years	103 (63.2%)
Duration of symptoms, months, mean ± SD		8.4 ± 3.6 (range: 3-37 months)
Duration of symptoms range	>12 months	56 (34.4%)
	<12 months	107 (65.6%)
Affected foot, right/left/bilateral		77/65/21 (47.2%, 39.9%, 12.9%)
Occupational status	Unemployed	55 (33.7%)
	On-site workers	73 (44.8%)
	Office workers	35 (21.5%)
Residential status	Rural	85 (52.1%)
	Urban	78 (47.9%)
Educational status	Illiterate	47 (28.8%)
	10^th^ grade or less	34 (20.9%)
	Above 10^th^ grade	82 (50.3%)
Success of treatment	Achieved	131 (80.3%)
	Not achieved	32 (19.7%)

Results of the paired-t test indicated that there is a significant large difference between the VAS pain intensity score before (8.6 ± 0.6) and after (2.2 ± 1.8) the PRP injection (t(162)=41.8; p<0.001). The mean difference in VAS score before and after intervention was 6.4 ± 1.9 after 12 weeks (Table 2).

**Table 2:** VAS pain scores before and after the treatment with PRP injection Paired t-test was used with a p-value ≤0.05 deemed significant VAS: Visual Analog Scale, PRP: platelet-rich plasma, SD: standard deviation

	Mean ± SD	p-value	Test statistic (t)
VAS before treatment (all patients); n=163	8.6 ± 0.6	<0.001	41.8
VAS after treatment (all patients); n=163	2.2 ± 1.8		
Mean difference; n=163	6.4 ± 1.9		
VAS before treatment (successful treatment); n=131	8.6 ± 0.3		
VAS after treatment (successful treatment); n=131	1.5 ± 1		
VAS before treatment (unsuccessful treatment); n=32	8.6 ± 0.6		
VAS after treatment (unsuccessful treatment); n=32	5.3 ± 1.3		

Chi-square analysis revealed a higher likelihood of successful treatment for those with symptoms less than 12 months (p≤0.05). No other variables (gender, age group, residence, literacy) significantly impacted treatment efficacy (all p>0.05) (Table 3).

**Table 3:** Treatment effect by gender, duration of disease, residential status, age group, literacy and occupational status Chi-square test was used with a p-value ≤0.05 deemed significant; χ²: chi-square

Characteristics	Treatment success		p-value	χ²
	Yes (n=131)	No (n=32)		
Gender				
Male (n=64)	51 (38.9%)	13 (40.6)	0.860	0.031
Female (n=99)	80 (61.1%)	19 (59.4%)		
Duration of disease				
<12 months (n=107)	91(69.5%)	16 (50 %)	0.038	4.321
>12 months (n=56)	40(30.5%)	16 (50%)		
Residential status				
Rural (n=85)	67 (51.1%)	18 (56.3%)	0.604	0.269
Urban (n=78)	64 (48.9%)	14 (43.4%)		
Age groups				
<50 years (n=103)	82 (62.6%)	21 (65.6%)	0.750	0.102
>50 years (n=60)	49 (37.4%)	11 (34.4%)		
Literacy				
Literate (n=116)	91 (69.5%)	25 (78.1%)	0.332	0.940
Illiterate (n=47)	40 (30.5%)	07 (21.9%)		
Occupational status				
Unemployed (n=55)	45 (34.4%)	10 (31.3%)	0.944	0.115
On-site workers (n=73)	58 (44.3%)	15 (46.9%)		
Office workers (n= 35)	28 (21.4%)	07 (21.9%)		

## Discussion

Plantar fasciitis is one of the leading causes of heel pain [1,2], attributed to its various etiological factors [6,7]. Despite numerous treatment options, unsatisfactory outcomes often prompt patients to seek more effective modalities [8,10]. PRP emerges as a novel approach in plantar fasciitis treatment. This study aimed to assess the effectiveness of autologous PRP injection for plantar fasciitis, clinically evaluating outcomes within a 12-week post-injection period. Our findings revealed significant clinical improvement in VAS pain intensity scores without any reported complications by the end of the 12-week follow-up period.

PRP injections promote healing in plantar fasciitis, likely due to the presence of growth factors and cytokines stored in platelet granules [14]. These factors, including platelet-derived growth factor, platelet-derived endothelial growth factor, transforming growth factor (TGF)-β1, interleukin (IL)-4, IL-8, IL-13, IL-17, tumor necrosis factor-alpha, and interferon-alpha, trigger key processes for tissue repair and regeneration [12,15,16]. They stimulate fibroblast activity (migration and proliferation), enhance blood vessel formation (vascularization), increase collagen production, and remove cellular waste (debris). Notably, TGF-β1 specifically increases the production of type I collagen, a crucial component of healthy tendons [12].

Sheth et al. [14] investigated the effectiveness of autologous PRP for orthopedic indications. They concluded that the evidence supporting the increasing clinical use of PRP for orthopedic bone and soft tissue injuries remains uncertain. This uncertainty is likely due to inconsistencies in study protocols, platelet separation techniques, and outcome measures. In contrast, our study on the use of autologous PRP for plantar fasciitis demonstrated a treatment success rate of over 80%.

Studies have shown PRP injections can be effective in reducing plantar fascia thickness and pain [17]. However, the effectiveness may vary depending on how the PRP is prepared. This includes factors like the centrifugation technique, what activates the platelets, the number of platelets in the final product, and even the presence of white blood cells [18]. Centrifugation is a common technique that concentrates platelets in the blood several times. In our study, we used a double centrifugation process (first spin at 200 g for 10 minutes, then another spin at 300 g for 10 minutes). Similar to other research on plantar fasciitis, this double spin method likely resulted in a platelet concentration six to eight times greater than normal blood [12]. That study also showed a significant reduction in pain scores (VAS score from 9.1 to 1.6) and an 88% patient satisfaction rate, along with a decrease in plantar fascia thickness.

In our study, we chose calcium gluconate to activate the platelets in the PRP. This selection offered advantages over other activators, such as calcium chloride and bovine thrombin. Calcium gluconate avoids the risks associated with thrombin, a common activator known to cause complications [19]. Additionally, it's easier to sterilize compared to calcium chloride. Choosing calcium gluconate for platelet activation provided several benefits. It allows for a slow release of growth factors over roughly 24 hours, starting low and reaching a peak at the optimal time for healing [20]. Furthermore, it delays blood clot formation and avoids the potential for antibody formation, a concern with bovine thrombin [20,21].

The final platelet concentration in autologous PRP is directly influenced by the patient's whole blood platelet count. Individuals with low platelet counts will consequently have lower PRP concentrations, leading to reduced growth factor release [12,22]. To ensure adequate platelets in the post-centrifugation plasma, only patients with platelet counts exceeding 250/μL were included in this study. Although the double-spin centrifugation technique is expected to yield a four- to sixfold increase in platelet concentration [12,22], precise measurement of the final PRP's platelet count is essential to confirm adequate growth factor release.

While local anesthetics can negatively impact PRP treatment, our study opted to forego them entirely to avoid these potential drawbacks [23-25]. These drawbacks include an acidic environment detrimental to treated cells and potential overlapping effects with PRP's mechanism of action [23,24]. Encouragingly, research suggests local anesthetics do not hinder growth factor release [25]. Interestingly, despite the absence of anesthesia, none of our patients reported significant discomfort during or after the procedure.

Our study evaluated the effects of a single PRP injection for plantar fasciitis. Several studies have investigated similar injection patterns [12,17]. There is no consensus in the available literature regarding the optimal frequency of initial therapy. At present, a single injection technique appears plausible. An alternative approach might be to use a single injection initially, followed by a repeat injection if symptoms recur after an initial positive response, as suggested by Scioli [26].

Our study found that patients receiving autologous PRP injections within 12 months of symptom onset experienced a higher success rate compared to those treated later. The authors postulate that this timeframe aligns with the potential for reduced responsiveness in chronically inflamed fascia to the growth factors released by platelets, although further research is needed to confirm this mechanism. A comprehensive literature search yielded no prior data supporting this specific correlation.

Our study has several limitations. First, the lack of standardized PRP concentration led to variations in the number of platelets injected among patients, hindering our ability to correlate platelet count with treatment efficacy. Second, we did not offer a second PRP injection to patients who failed to respond to the initial therapy, as our primary focus was on assessing the effect of a single autologous PRP injection. Third, this study cannot determine the duration of PRP effectiveness. Further research is needed to establish proper protocols and the duration of their effectiveness. This study benefited from several strengths. First, we employed a standardized technique for PRP extraction, ensuring consistency across procedures. Second, we established a minimum platelet count of >250/μL in whole blood to ensure adequate platelet availability in the PRP. Finally, patients abstained from medications like NSAIDs throughout the study period except for the first three days, eliminating potential interference with treatment.

This study investigated the effectiveness of autologous PRP injections for plantar fasciitis. Patients experienced significant clinical improvement within 12 weeks without complications, likely due to the healing properties of PRP's growth factors. The study highlights the advantages of using a double centrifugation technique with calcium gluconate as an activator for optimal PRP preparation. While limitations include a lack of standardized platelet concentration and a comparison group, the findings suggest PRP's potential as a valuable treatment option for plantar fasciitis.

## Conclusions

This study suggests that early intervention with autologous PRP injections may be a promising non-invasive and cost-effective treatment for plantar fasciitis. Our findings demonstrate significant pain reduction (measured by VAS scores), especially in patients treated early after symptom onset. While these results are encouraging, further research is needed to validate their efficacy and explore suitable patient selection criteria. The safety and potential cost-effectiveness of PRP compared to traditional treatments also warrant further investigation. If these findings are confirmed, PRP injection could become a valuable tool for managing plantar fasciitis in clinical practice.
